# Treatment of colorectal liver metastases in Germany: a ten-year population-based analysis of 5772 cases of primary colorectal adenocarcinoma

**DOI:** 10.1186/1471-2407-14-810

**Published:** 2014-11-04

**Authors:** Christina Hackl, Peter Neumann, Michael Gerken, Martin Loss, Monika Klinkhammer-Schalke, Hans J Schlitt

**Affiliations:** Department of Surgery, University Hospital Regensburg, Franz-Josef Strauss Allee 11, 93053 Regensburg, Germany; Tumour Centre Regensburg, Josef-Englert-Straße 9, 93053 Regensburg, Germany

**Keywords:** Metastasis, Liver resection, Colon cancer, Hepatic surgery

## Abstract

**Background:**

Purpose of this study was to analyse the surgical management and long-term clinical outcome of patients diagnosed with colorectal liver metastases (CLM) over a period of 10 years using data from a German tumour registry.

**Methods:**

Retrospective analysis of 5772 patients diagnosed with colorectal adenocarcinoma between 2002 and 2007. Follow-up was continued until 2012.

**Results:**

1426 patients (24.7%) had CLM; 1019 patients (71%) had synchronous, 407 patients (29%) developed metachronous CLM. Hepatic resection was performed in 374 of the 1426 CLM patients (26%). A significant increase in liver resection rate from 16.6% for the 2002 cohort to 32% in later cohorts was observed. In centers specialized in liver surgery, CLM resection rates reached 46.6%. However, up to 52% of patients diagnosed with three or less CLM did not undergo liver surgery, although, if resected, patients with 1 CLM show a similar long-time survival as CRC patients who do not develop any CLM. Univariate and multivariate analyses adjusted for age, sex, year of resection, time of CLM diagnosis and number of CLM revealed a significant survival benefit for CLM resection (HR =0.355; CI 0.305-0.414). Furthermore, significant impact on OS was seen for age at diagnosis, perioperative chemotherapy and number of CLM.

**Conclusions:**

We here present the first long-term, population-based analysis of the surgical management of CLM in Germany. Significant increase in hepatic resection rates, translating to a significant benefit in OS, was seen over years. However, we still see a striking potential for further improvements in interdisciplinary CLM management.

**Electronic supplementary material:**

The online version of this article (doi:10.1186/1471-2407-14-810) contains supplementary material, which is available to authorized users.

## Background

Medical and surgical treatment of advanced metastatic colorectal cancer (CRC) has undergone enormous improvement during the last years and is still evolving. Accounting for 12.2% of all cancer-related deaths in Europe, the majority of patients diagnosed with metastatic CRC present with unresectable metastatic disease [1]. In contrast, surgery remains the only curative approach to colorectal liver metastases (CLM) and liver resection is indicated in all “resectable” patients [2]. Definition of resectabiliy however – including “manageable” margin-negative resection, “adequate” residual perfusion and biliary drainage as well as a minimum of 20% residual healthy liver, remains vague and necessitates interdisciplinary evaluation by expert oncologists, surgeons and radiologists [1, 3]. Furthermore, perioperative chemotherapy has been shown to facilitate surgery in extended metastatic disease, to increase resectability of initially unresectable CLM by 7-40%, and to increase progression-free survival in resectable CLM [1]. Single centre publications have shown CLM resection rates of 20-45% and 5-year survival rates after CLM resection of up to 64% [1–6]. However, single, high-volume academic centres represent highly selected patients and the results do not always reflect the clinical reality. In a first population-based study of CLM patients in Southern Germany, we had analysed 884 patients diagnosed with CRC in 2002. An overall CLM resection rate of 19.1% was described; a higher resection rate (28.3%) was observed in a subgroup of patients treated in centres specialized in liver surgery. However, a relevant undertreatment of CLM patients was seen [7]. We therefore decided to analyse the evolution of CLM management over a period of ten years. The present study is the first long-term analysis of 5772 patients diagnosed with colorectal adenocarcinoma between 2002 and 2007 in Germany. Observation was continued until 2012, resulting in a minimum follow-up of 5 years (maximum 10 years).

## Methods

### Patient population and data acquisition

For the present analysis, we included all patients that were diagnosed with colorectal adenocarcinoma between 2002 and 2007 registered in the Tumorzentrum Regensburg (tumor center Regensburg). This center collects epidemiological and clinical information on all cancer patients in the Southern German Regions of Upper Palatinate and Lower Bavaria. These regions have a total population of approximately two million inhabitants. Data was collected from standardised cancer report sheets submitted from care centres and oncologists as well as from archived hospital discharge letters for each patient. All diagnoses were confirmed by histology. Life-status of the patients was ascertained using death-certificates and information from the registration offices of the patients’ respective resident districts. The observation time was the interval between diagnosis of primary tumour until last follow up or death of the patient. Cut-off date was December 2012. Patients suffering from more than one tumour entity, i.e. another malignancy in addition to colorectal cancer, were excluded. Data pertaining to demographics, TNM staging, grading, histology, completeness of resection, adjuvant treatment, localisation, time and characteristics of metastases, surgical interventions, outcomes and type of treatment institution (centre specialized in liver surgery or regional hospital) were reviewed for each patient and entered into a database. The study population includes a total of 5772 patients with complete clinical records, representing a completeness of 85%. Synchronous metastasis was defined as a lesion documented simultaneously or within 3 months of the primary diagnosis – under the consideration that a real hepatic tumour “recurrence” that early after primary treatment is very unlikely. A diagnosis of metastases later than 3 months after the primary was defined as metachronous metastasis. All collection and retrospective analysis of patient information was anonymized, in accordance with the Declaration of Helsinki, and approved by the Bavarian Law of Cancer Registration. Permission for data analysis was obtained from the legal and ethical guarantors of the Regensburg tumor center.

### Statistical analysis

Overall survival (OS) time was censored at the time of death or last follow-up with a cut-off date in December 2012 in order to have a minimum follow-up of 5 years for each patient. Maximum follow-up, i.e. patients diagnosed with CRC in the year 2002, was 10 years. Median follow-up was 7.1 years after diagnosis of the primary (6.7 years after CLM diagnosis). Survival curves were estimated by the Kaplan-Meier method. Hazard Ratios for OS were estimated by Cox-Proportional-Hazard-Regression. Results were considered significant at p <0.05. All statistical and descriptive analyses were performed using SPSS software, version 19.0.

## Results

### Study population and occurrence of hepatic metastases

**Figure 1:**
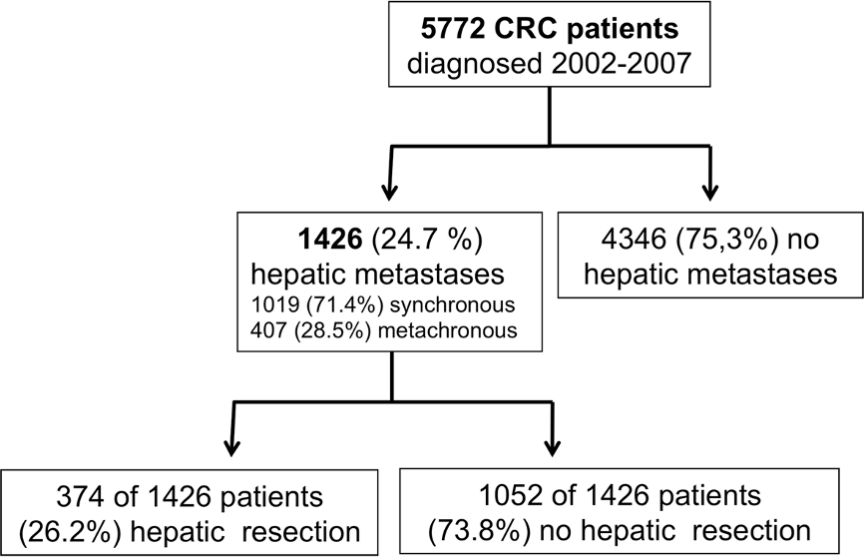
**Synopsis of patients analysed in the present study.** A total of 5772 patients diagnosed with colorectal cancer (CRC) in the years 2002–2007 were included. Of these, 1426 patients developed hepatic metastases and 374 underwent hepatic resection.

**Figure 2:**
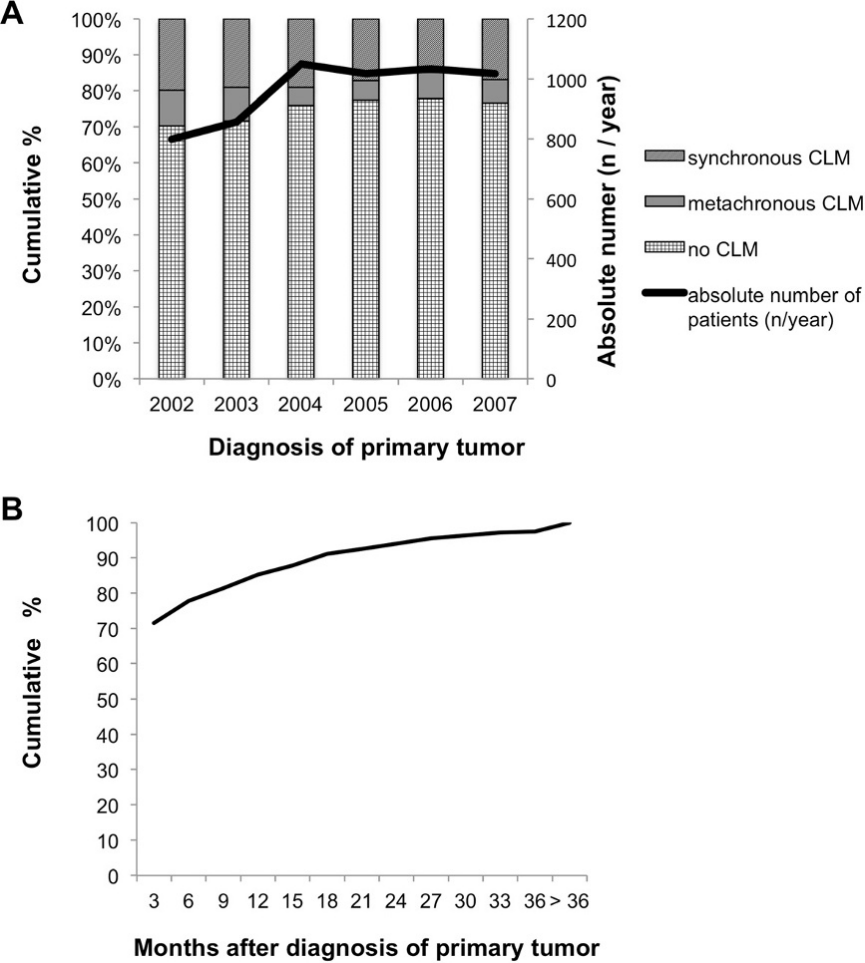
**Incidence of colorectal cancer in the study population. A** Relative (left axis) and absolute numbers (right axis, black line) of colorectal cancer patients with no (chequered), and metachronous (grey), synchronous (hatched) colorectal liver metastases by year of CRC diagnosis. **B** Relative cumulative incidence of colorectal liver metastases in months after diagnosis of the primary tumour.

A total of 5772 patients, diagnosed with colorectal cancer (CRC) between January 2002 and December 2007, were included into the present analysis (see Figure 1). One thousand four-hundred and twenty-six patients (=24.7%) developed hepatic metastases. Synchronous metastases were diagnosed in 1019 patients (71.4% of CLM patients and 17.65% of all patients), 407 patients (28.5% of CLM patients and 7.05% of all patients) developed metachronous metastases. These relations and the absolute numbers of patients diagnosed with CRC and CLM per year is shown in Figure 2A. While 799 and 856 CRC patients were documented in the years 2002 and 2003, more than 1000 patients each year were documented in 2004–2007. Rates of synchronous and metachronous metastases remained constant in the above mentioned range. Of all CLM, 85.3% were diagnosed within 12 months, 94.0% within 24 months and 97.5% within 36 months after diagnosis of the primary CRC (see Figure 2B). In all cases, CLM diagnosis was based on ultrasound plus computed tomography and/or magnetic resonance imaging.

Table 1 summarizes the characteristics of all 5772 patients, comparing demographic data and primary tumour staging of patients diagnosed with CLM and patients without CLM. Among the CLM patients, 61.4% were male and 38.6% female. Mean age at primary tumour diagnosis was 67 years (median 68 years). The majority of primary tumours were located in the colon (62.8%), 5.3% at the colorectal junction and 31.9% in the rectum. All these parameters were not significantly different in CRC patients with or without CLM. Initial UICC staging was significantly higher in CLM patients. However, 9.1% of CRC patients not diagnosed with hepatic metastases initially had an UICC stage IV at primary diagnosis. Location of non-hepatic distant metastases in these patients is shown in Additional file 1: Table S1.

**Table 1:** **Characteristics of 5772 patients with or without colorectal liver metastases (CLM)**

		CLM			
		Yes		No	
		n	%	n	%
**Sex**	**Male**	876	61.4	2530	58.2
	**Female**	550	38.6	1816	41.8
**Age at CRC diagnosis**	**< 30**	3	0.2	6	0.1
	**31-40**	13	0.9	53	1.2
	**41-50**	100	7.0	226	5.2
	**51-60**	225	15.8	646	14.9
	**61-70**	475	33.3	1313	30.2
	**71-80**	458	32.1	1462	33.6
	**>80**	152	10.7	640	14.7
**Primary**	**Colon**	896	62.8	2573	59.2
	**CR junction**	75	5.3	230	5.3
	**Rectum**	455	31.9	1543	35.5
**initial UICC stage**	**I**	23	1.6	1178	27.1
	**II**	74	5.2	1368	31.5
	**III**	153	10.7	1406	32.4
	**IV**	1176	82.5	394	9.1
**Total**		**1426**	**100**	**4346**	**100**

The characteristics of CLM patients, comparing those who underwent hepatic resection with patients who did not undergo liver surgery, are presented in Additional file 2: Table S2. Among resected CLM patients, 63.6% were male and 36.4% female. Mean age at primary diagnosis was 64 years (median 65 years). The majority of primary tumours (57.2%) was located in the colon, 7.2% at the colorectal junction and 35.6% in the rectum. Synchronous metastasis had been present in 67.6% of liver resected patients. All of these parameters were not significantly different to CLM patients not undergoing liver resection, except for age at diagnosis, which was 5 years higher in patients without resection. UICC class IV was significantly higher in CLM patients not undergoing liver surgery, indicating a higher rate of non-hepatic metastasis.

### Surgical approach to colorectal liver metastases

**Figure 3:**
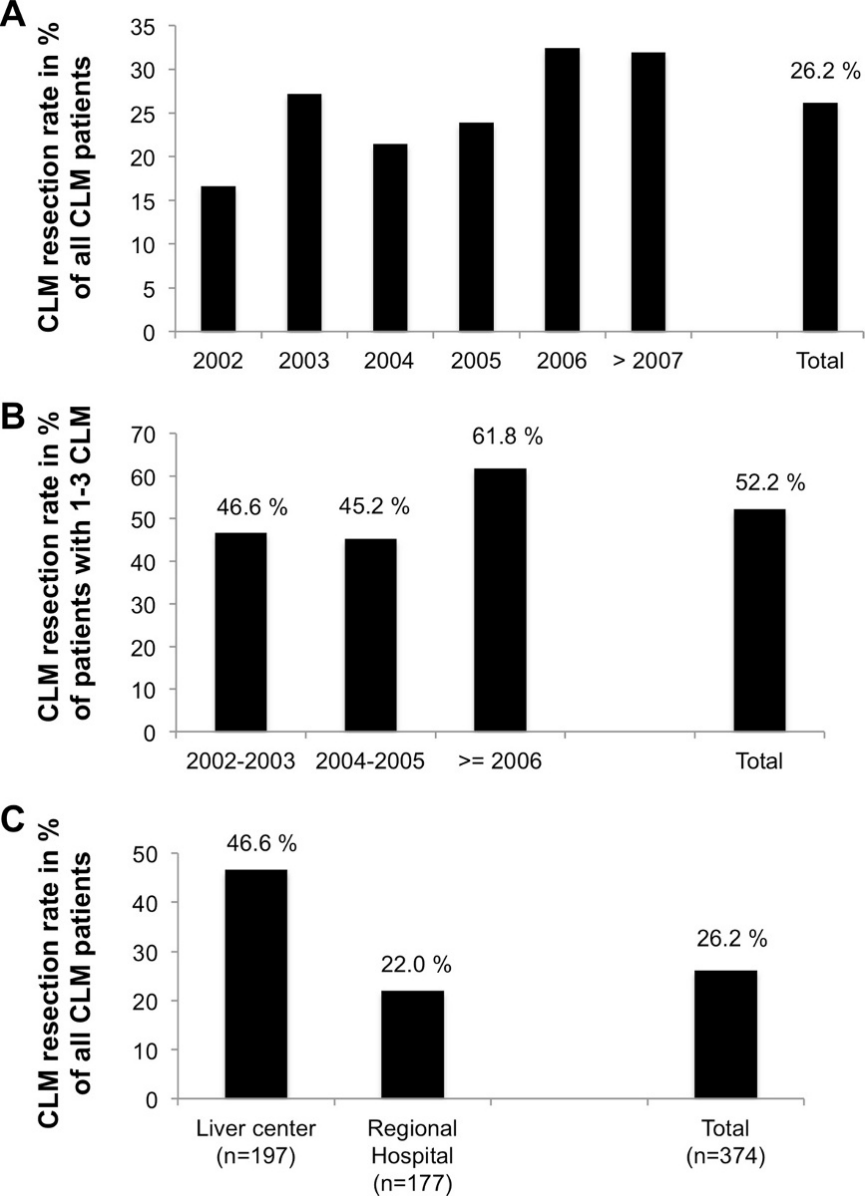
**Resection rates of colorectal liver metastases. A** Resection rate of colorectal liver metastases (CLM) in % by year of CLM diagnosis. **B** Resection rate of CLM by year of CLM diagnosis in a subgroup of patients diagnosed with 1–3 CLM. **C** Resection rate of CLM by treatment centre: 2 academic liver centres compared with 28 regional hospitals.

Of 1426 patients diagnosed with CLM, 383 (26.8%) patients were documented to have 3 or less hepatic metastases and 729 (51.1%) patients showed more than 3 metastases. The number of hepatic lesions was not documented in 314 patients (22%, see Additional file 3: Table S3). These proportions remained comparable throughout all cohorts (see Additional file 4: Table S4). The surgical approach chosen is shown in Additional file 3: Table S3, with the majority of patients undergoing atypical liver resections. In total, 374 of the 1426 CLM patients underwent hepatic resection in curative intent, representing an overall resection rate of 26.2%. Hepatic resection rates increased over time from 16.6% in 2002 to >21% in the years 2003–2005 (2003: 27.2%; 2004: 21.5%; 2005: 23.9%) to >30% after 2005 (2006: 32.4%; 2007 and later: 31.9%, see Figure 3A). A subgroup analysis, only taking into account CLM patients with three or less hepatic metastases, showed an overall average resection rate of 52.2%, increasing from 46.6% in 2002–2003 to >61% after 2005 (see Figure 3B). Hepatic resections were performed in a total of 30 hospitals; including 2 centres specialized in liver surgery. In 197 of 374 patients (=52.7%) undergoing liver resection, hepatic surgery was performed in one of these two centres and an overall resection rate of 46.6% was documented, compared to an overall resection rate of 22.0% in CLM patients treated in regional hospitals (see Figure 3C).

### Long-term clinical outcome

**Figure 4:**
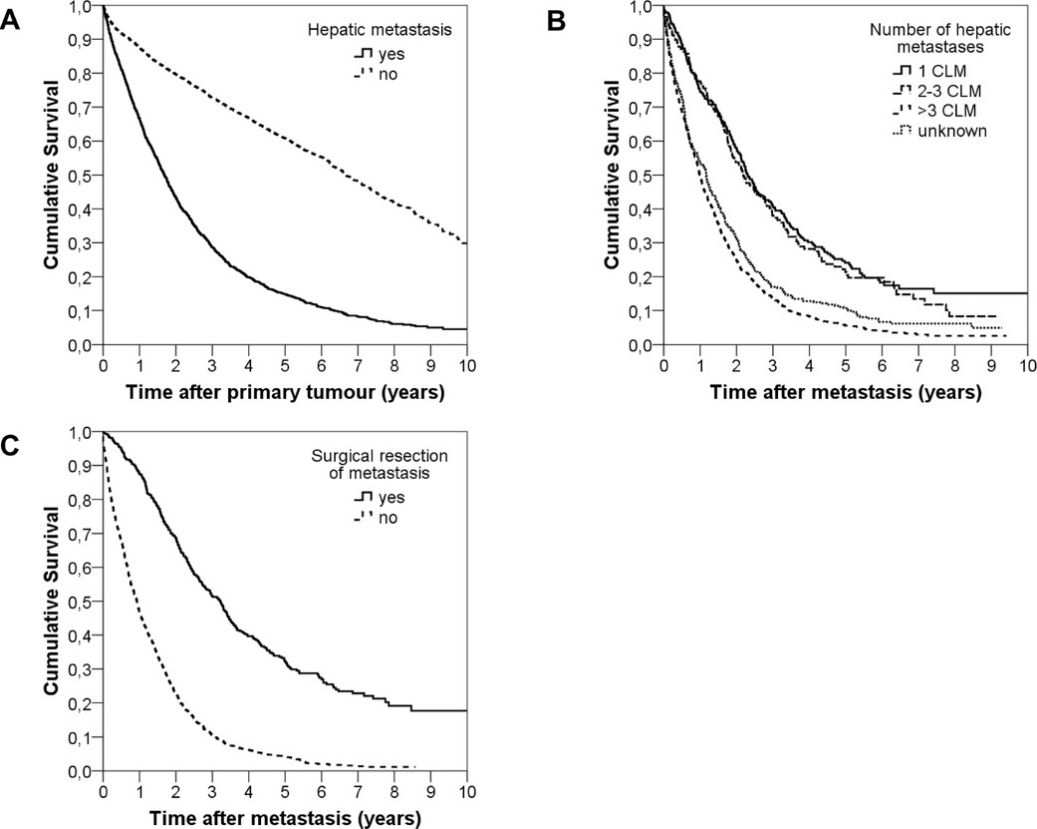
**Ten year overall survival analyses. A** Comparison of 10-year OS in colorectal cancer cases with (n = 1426) an without (n = 4346) liver metastases (CLM) 2002–2007. **B** Comparison of 10-year OS in colorectal cancer liver metastases (CLM) patients by number of CLM independent of CLM resection. (1CLM: n = 236; 2-3CLM: n = 147; >3CLM: n = 729; unknown number of CLM: n = 314). **C** Comparison of 10-year OS in colorectal cancer liver metastases (CLM) patients with (n = 374) and without (n = 1052) curative liver resection.

**Figure 5:**
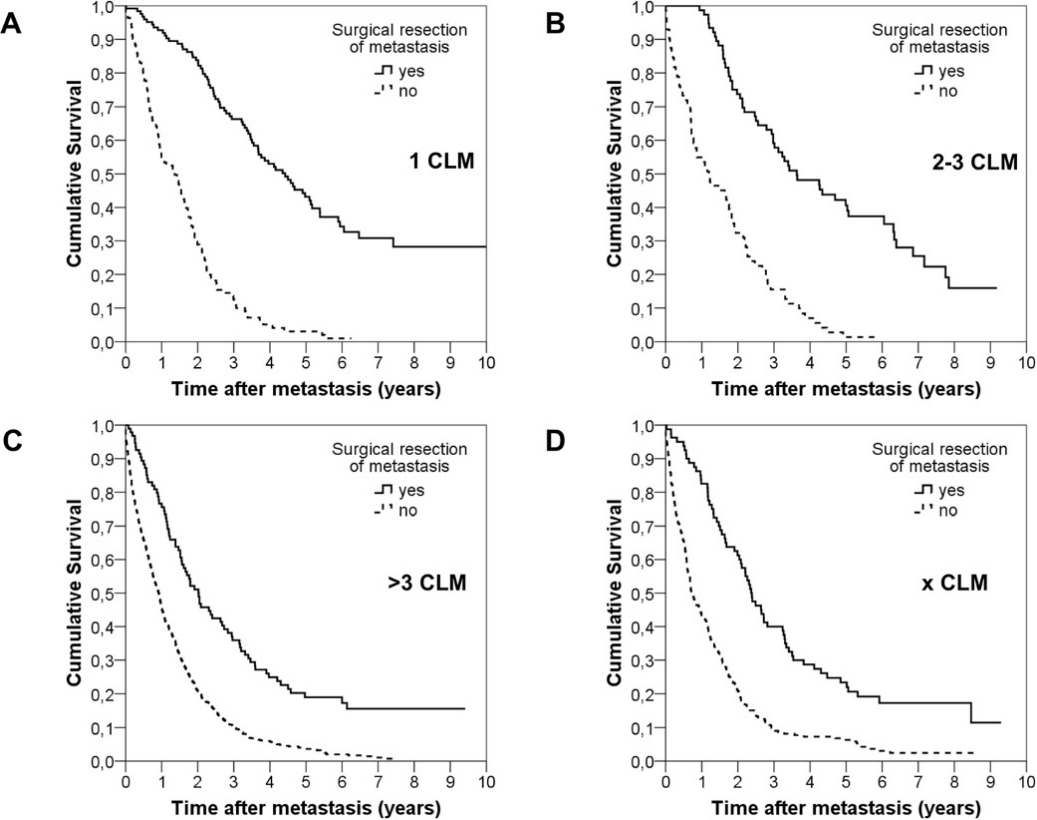
**Ten year overall survival analyses in patients undergoing hepatic resection. A** Comparison of 10-year OS - Liver resection yes (n = 124) versus no (n = 112) in patients with singular metastasis. **B** Comparison of 10-year OS - Liver resection yes (n = 76) versus no (n = 71) in patients with 2–3 metastases. **C** Comparison of 10-year OS - Liver resection yes (n = 94) versus no (n = 635) in patients with more than 3 metastases. **D** Comparison of 10-year OS - Liver resection yes (n = 80) versus no (n = 234) in patients with unknown (x CLM) number of metastases.

Kaplan Meier curves analysing the 10-year OS are shown in Figures 4 and 5, results for 1-, 2-, 5- and 10-year survival are shown in Table 2. CRC patients diagnosed with CLM showed a significantly lower 10-year OS compared to CRC patients without liver metastases (4.6% versus 29.8%, respectively, median survival 1.7 versus 6.3 years, p <0.001, Figure 4A and Table 2). Survival analyses in relation to the number of hepatic metastases are shown in Figure 4B and Table 2. Patients with three or less CLM had a significantly longer median survival than patients with more than three or unknown number of liver metastases (median survival 2.3 years versus 1.0 years, p =0.02). 10-year OS was 15.1% in patients with 1 CLM versus 2.6% in patients with more than three CLM, irrespective of hepatic resection. CLM patients who underwent hepatic resection showed a median survival of 4.3 years versus 1.9 years in CLM not undergoing liver resection (p <0.001), translating into a 5-year and 10-year OS of 32.2% and 17.6% versus 4.0% and 1.1%, respectively (see Figure 4C, Table 2). This significant benefit of hepatic resection was especially pronounced in patients with 3 or less metastases (5-year OS 40.6% versus 1.4%, p <0.001), but remained significant also for patients diagnosed with multiple or unknown number of hepatic metastases (see Figure 5, Table 2). Notably, only 52% of patients diagnosed with a single liver metastasis underwent hepatic resection (see Additional file 5: Table S5, Figure 3B). This finding is of particular importance, as patients with a single CLM who undergo hepatic resection show a similar long-time survival as CRC patients who do not develop any CLM (10-year OS in resected 1 CLM patients 28.3%, in CRC patients with no CLM 29.8%, n.s.; Table 2).

**Table 2:** **Synopsis of overall survival rates**

		Overall survival rates in %					
Patients	Group	Number	1-year	2-year	5-year	10-year	Log-rank
**CRC**	**CLM**	1426	66.7	43.4	14.8	4.6	< 0.001
	**No CLM**	4346	87.6	79.6	60.9	29.8	
**CLM**	**CLM resection**	374	87.4	68.7	32.2	17.6	< 0.001
	**No CLM resection**	1052	46.6	22.5	4.0	(1.1)	
**CLM**	**1 CLM**	236	74.5	57.8	24.1	15.1	
	**2-3 CLM**	147	77.6	53.7	21.3	(8.4)	
	**>3 CLM**	729	49.3	24.8	5.6	(2.6)	
	**x CLM**	314	53.4	31.3	10.7	(5.0)	
**1 CLM**	**CLM resection**	124	92.7	83.8	43.2	28.3	< 0.001
	**No CLM resection**	112	54.9	28.9	3.1	(1.0)	
**2-3 CLM**	**CLM resection**	76	98.7	73.7	40.6	(15.9)	< 0.001
	**No CLM resection**	71	54.9	32.4	1.4	(1.4)	
**>3 CLM**	**CLM resection**	94	75.5	51.1	18.9	(15.5)	< 0.001
	**No CLM resection**	635	45.4	20.9	3.6	(0.7)	
**x CLM**	**CLM resection**	80	82.5	61.3	23.3	(11.5)	< 0.001
	**No CLM resection**	234	43.4	21.0	6.3	(2.4)	
**CLM**	**CLM diagnosis 2002-2004**	648	55.9	33.5	10.2	4.7	0.231
	**CLM diagnosis >= 2005**	778	58.5	35.6	12.5	(7.3)	

In a subgroup, we analysed whether patients diagnosed with CLM in 2005 and later years showed a difference in long-term-survival compared to patients diagnosed with CLM before 2005. A trend toward an improved survival was seen (Additional file 6: Figure S1, not significant).

Perioperative chemotherapy had been given in 180 patients (48%) undergoing liver surgery for CLM. Chemotherapeutic regimens were manifold, ranging from 5-FU monotherapy to triple chemotherapy plus targeted therapy. In the present analysis, no significant influence of specific chemotherapy regimen on resection rate or clinical outcome was seen in univariate analysis (data not shown). However, multivariate analysis adjusted for age, sex, year of liver resection, time of CLM after diagnosis of the primary tumour and number of hepatic lesions, showed a significant benefit on overall survival for perioperative chemotherapy for CLM (hazard ratio 0.606, 95%-CI 0.540-0.681, see Table 3).

**Table 3:** **Multivariate analysis of factors influencing overall survival**

Multivariate cox regression	p	HR	CI
(Overall survival)			
Resection (yes vs no) - unadjusted	< 0.001	0.306	0.266 - 0.351
Resection (yes vs no) - adjusted	< 0.001	0.355	0.305 - 0.414
Age	< 0.001	1.017	1.012 - 1.023
Sex (female vs male)	0.298	1.062	0.948 - 1.189
Year of metastasis	0.879	1.003	0.970
Synchronous vs Metachronous	0.799	0.983	1.036
Number of CLM 1		1.000	
Number of CLM 2-3	< 0.001	1.067	0.864 - 1.119
Number of CLM >3	0.576	1.659	0.850 - 1.340
Number unknown	< 0.001	1.430	1.387 - 1.985
Chemotherapy (yes vs no)	< 0.001	0.606	0.540 - 0.681

Univariate and multivariate analysis, adjusted for age, sex, year of resection, time after primary tumour and number of metastasis, revealed significant OS benefit for CLM resection (HR =0.355).

In multivariate analysis, significant impact on OS was seen for age at diagnosis, number of CLM, and chemotherapeutic treatment. No significant influence on OS was seen for the parameters sex, year of resection, time of CLM diagnosis after primary tumour (metachronousvs synchronous). p = Level of significance, HR = Hazard ratio, CI =95% confidence interval.

In multivariate analysis, the parameter “hepatic resection” translated into a significant benefit on OS (hazard ratio 0.355, 95%-CI 0.305-0.414). Significant impact on OS was also seen for age at diagnosis and number of CLM. No significant influence on OS was seen for the parameters sex, year of resection, and time of CLM diagnosis after primary tumour (metachronous vs synchronous, see Table 3).

## Discussion

Surgical resection represents the only potentially curative approach to colorectal liver metastases [1, 2]. During the past years, enormous improvements in both, surgical technique and perioperative chemotherapeutic treatment options, have increased CLM resection rates to 20 - 45% and five-year survival after hepatic resection to up to 64% in selected, single-centre analyses [2]. However, these results are obtained from highly selected and ideally monitored patients and do not reflect clinical reality. Therefore, we decided to analyse the surgical management and long-term clinical outcome of CLM patients using data from a German tumour registry over a period of ten years.

In the present study, a total of 5772 patients diagnosed with CRC were analysed. In 2002 and 2003, respectively 799 and 856 patients with complete records were analysed, representing a completeness of 80%. Increased and constant numbers of patients analysed in later years do represent a completeness rate of >90%. CLM were diagnosed in 24.7% of CRC patients. This rate is at the lower range as compared to previously published population-based analyses as well as single-centre publications [3, 5, 6, 8–13]. Furthermore, our rate of synchronous metastases is at the higher range as compared to other population-based studies using the same definition [5, 8–13]. We therefore assume a slight deficit in completeness of documented metachronous CLM in our tumour registry. 94% of CLM are diagnosed within 24 months of primary diagnosis, explaining the constant CLM rates until 2009, i.e. 24 months after study entry of the last patients.

Although definition of resectability does no longer include number of hepatic metastases, we assume that the majority of patients diagnosed with 3 or less CLM would be considered to be resectable. Surprisingly, only 52.2% of these patients underwent liver resection, indicating a high potential for further improvement in surgical therapy in this subgroup. However, a significant increase in resection rate in this subgroup from 46.6% in 2002/2003 to 61.8% in 2006 and later years was observed. Overall resection rate of CLM was 26.2%, which compares favourably to other population-based as well as single-centre analyses [5, 8–13]. Over time, a significant increase in resection rates from 16.6% in the 2002 cohort to >21% in the 2003–2005 cohorts to >30% in cohorts after 2005 was observed. In 2006, certification of comprehensive colorectal cancer centres was initialized by the German Cancer Society. During the study period, 8 of all 30 hospitals being part of the present analysis have been certified as colorectal cancer centres. The increase in CLM resection rates may in part reflect the implication of standard operating procedures during interdisciplinary management of CRC patients treated in certified centres. These standards include mandatory discussion of the surgical options of each individual patient by comprehensive tumour-board meetings.

A subgroup of 423 CLM patients (29.7%) in the present study were treated in one of two academic centres specialized in liver surgery, which is a lower rate than in comparable population-based analyses [5, 7–13]. Of those 423 patients treated in academic centres, 197 (46.6%) underwent liver resection, compared to a resection rate of 22% in regional hospitals. The higher resection rate of patients treated in centres specialized for liver surgery is consistent with data published from large academic single-institution studies and may reflect selection and referral bias, but may also indicate that more resectable patients really underwent surgery in these two centres [1, 3]. Nevertheless, a resection rate of 46.6% is at the higher range as compared to selected, single-centre analyses [1, 2]. Together with the increase in hepatic resection rates over time in the present study as well as the number of still unresected CLM patients, especially in those patients diagnosed with three or less CLM, we here show for the first time a pronounced improvement in surgical management of CLM over time in Germany, but also a still striking potential for further increases in hepatic resection rates.

Kaplan-Meier analyses demonstrated – as expected - that patients without CLM had a significantly better 5-year and 10-year OS than CLM patients. In CLM patients, the number of metastases, irrespective of hepatic resection, was a significant factor for long-term OS. Long-term OS in CLM patients was significantly increased by liver resection, which was most pronounced in patients diagnosed with a singular hepatic lesion, but remained significant also for patients diagnosed with multiple CLM. Survival curves plateaued at 8–9 years after CLM diagnosis, thus being in line with the definition of “cure” from CLM published by Tomlinson and colleagues [14]. Cure rates from CLM of 17 to 25% have been published and ongoing trials evaluating perioperative triple-chemotherapy combined with targeted therapy for CLM patients report of 5-year survival rates of up to 64% [2]. Thus, our survival data are in line with previously published population-based and single-centre analyses.

Intraoperative ablative therapies in combination with curative resection have been applied in 24 patients of the present series (10 thermal ablations, 12 radiofrequency ablations, 2 cryotherapies). Previous studies have suggested that ablation of liver metastasis leads to more frequent and quicker liver recurrence [15]. Since the number of patients treated with a combined resection/ablation approach was very small, no subgroup analyses of recurrence-free or overall survival could be performed in the present series.

The timing of the resection of synchronous liver metastasis is a focus of international discussion. Some experts recommend simultaneously resecting the primary colorectal cancer and the CLM, others recommend staged resection with the primary tumor resected first and finally others recommend a “liver-first” approach with resection of the CLM first, followed by resecting the primary tumor in a following surgery [16]. In our series, 253 of 374 hepatic resections were performed in patients diagnosed with synchronous liver metastases. Of these 253 patients, 134 received resection of hepatic metastases and primary tumor during a simultaneous approach. In these cases, metastatic tumor burden could be controlled by atypical or segmental hepatic resection. Only 5 patients diagnosed with synchronous hepatic metastases underwent a “liver-first” approach, the remaining 114 patients received the “classic approach”, starting with the resection of the primary tumor. The rationale for this approach, e.g. bowel obstruction or bleeding caused by the primary tumor, remained unknown. Comparing the long-term clinical outcome of patients treated with the liver-first approach versus “classic approach” would be of high interest. Recent publications have discussed the liver-first approach as preferred treatment, since the long-term prognosis is more determined by controlling metastatic disease and hepatic resectability may be jeopardized by prior colorectal surgery [16]. Furthermore, neoadjuvant chemotherapy prior to liver-first hepatic resection may select patients benefiting most from curative surgery [16]. Due to the very limited number of liver-first resected patients, we cannot provide a statistically relevant result in this regard.

In the present study, perioperative chemotherapy was given in 180 CLM patients (48%) undergoing hepatic resection. However, chemotherapeutic regimens were manifold, ranging from 5-FU monotherapy to triple chemotherapy plus targeted therapy. Furthermore, timing of perioperative chemotherapy as neoadjuvant, adjuvant, or combined neo-/ and adjuvant regimens in relation to liver resection as well as total duration of chemotherapy was diverse and no significant influence of chemotherapy on resection rate or survival was seen in univariate analysis. Multivariate analysis however showed a significant benefit of perioperative chemotherapy for CLM. Perioperative chemotherapy for CLM is a highly relevant research focus and has been shown to increase resectability of initially unresectable CLM by 7-40% [1]. An international expert panel recommends chemotherapy for all patients diagnosed with unresectable CLM, followed by surgery as soon as resectability may occur [3]. The most efficient chemotherapy is presently studied in multiple randomized clinical trials. The CRYSTAL and OPUS trials showed significantly increased resection rates of CLM after treatment with Folfiri, respectively Folfox, combined with the targeted agent Cetuximab [17]. The CELIM trial, cross-comparing Folfox plus cetuximab versus Folfiri plus cetuximab, showed resection rates of CLM of 40-43% with no significant difference comparing both regimens [18]. Ongoing clinical trials evaluate the triple chemotherapeutic approach Folfoxiri combined with single or dual targeted agents [18–20]. Expert consensus recommends neoadjuvant chemotherapy in resectable CLM with more than one of the poor prognostic factors multiple metastases, size of CLM >5 cm, synchronous CLM, lymph-node positive primary CRC or high tumor markers [3].

We interpret our trend of an improved survival of CLM patients diagnosed after 2005 compared to CLM patients diagnosed 2002–2005 to be partly caused by the above mentioned CRC centre certification, partly caused by improved hepatic surgery and last but not least by advances in perioperative chemotherapy.

## Conclusions

In conclusion, the present study is the first large and long-term analysis of the incidence, the management and outcome of CLM patients in Germany. We show a pronounced increase in CLM resection over time. However, we also reveal a relevant surgical under-treatment of CLM. Certification of colorectal cancer centres after 2006, implicating region-wide standard operating procedures with interdisciplinary management of CRC patients, may in part explain the significant increase in CLM resection rates. However, there is still a striking potential for further improvements of interdisciplinary treatment of patients diagnosed with metastatic CRC.

## Electronic supplementary material

Additional file 1: Table S1: Localization of metastases in patients diagnosed with synchronous non-hepatic metastases (UICC stage IV, multiple locations possible). (TIFF 280 KB)

Additional file 2: Table S2: Characteristics of 1426 colorectal liver metastasis (CLM) patients with or without curative liver resection. (TIFF 462 KB)

Additional file 3: Table S3: Number of hepatic metastases and surgical management of 1426 patients with colorectal liver metastases. (TIFF 342 KB)

Additional file 4: Table S4: Number of hepatic metastases diagnosed over time. (TIFF 463 KB)

Additional file 5: Table S5: Hepatic resection rates in relation to number of colorectal liver metastases. (TIFF 278 KB)

Additional file 6: Figure S1: Comparison of 10-year OS in patients diagnosed with colorectal liver metastases with and without liver metastases before and after 2005: A trend towards an improved survival in patients diagnosed 2005 and later was seen (not significant). (PPTX 264 KB)

## Abbreviations

CI: Confidence interval
CLM: Colorectal liver metastases
CRC: Colorectal Cancer
OS: Overall Survival
UICC: Union for International Cancer Control.
