# Supplementary figures and images for: Treatment of colorectal liver metastases in Germany: a ten-year population-based analysis of 5772 cases of primary colorectal adenocarcinoma
Source: BMC Cancer. 2014 Nov 4;14:810. doi: 10.1186/1471-2407-14-810 (PMC4230526; doi:10.1186/1471-2407-14-810)

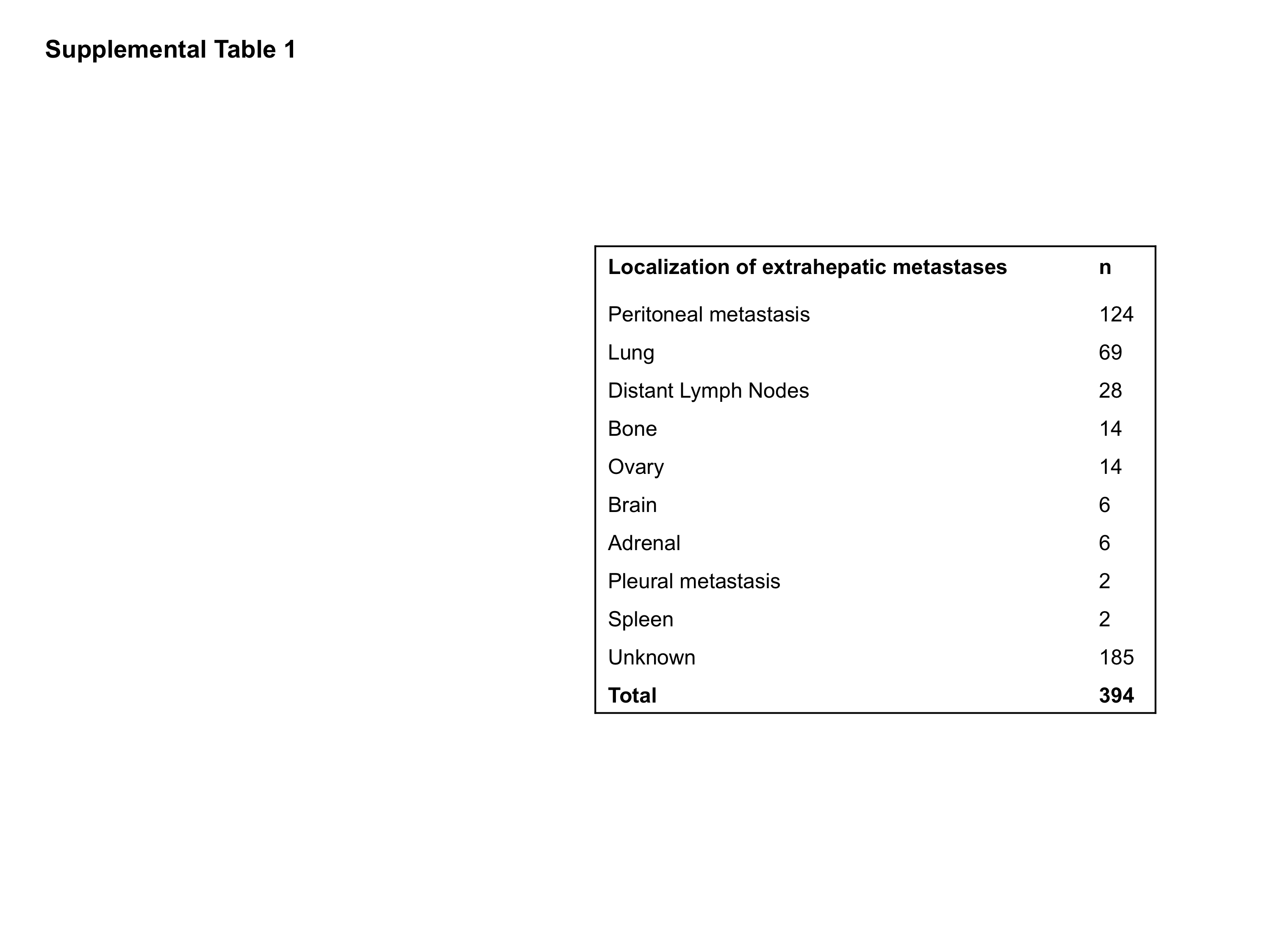

Supplement: Supplementary file 1 — Additional file 1: Table S1: Localization of metastases in patients diagnosed with synchronous non-hepatic metastases (UICC stage IV, multiple locations possible). (TIFF 280 KB) [file 12885_2014_4981_MOESM1_ESM.tiff]

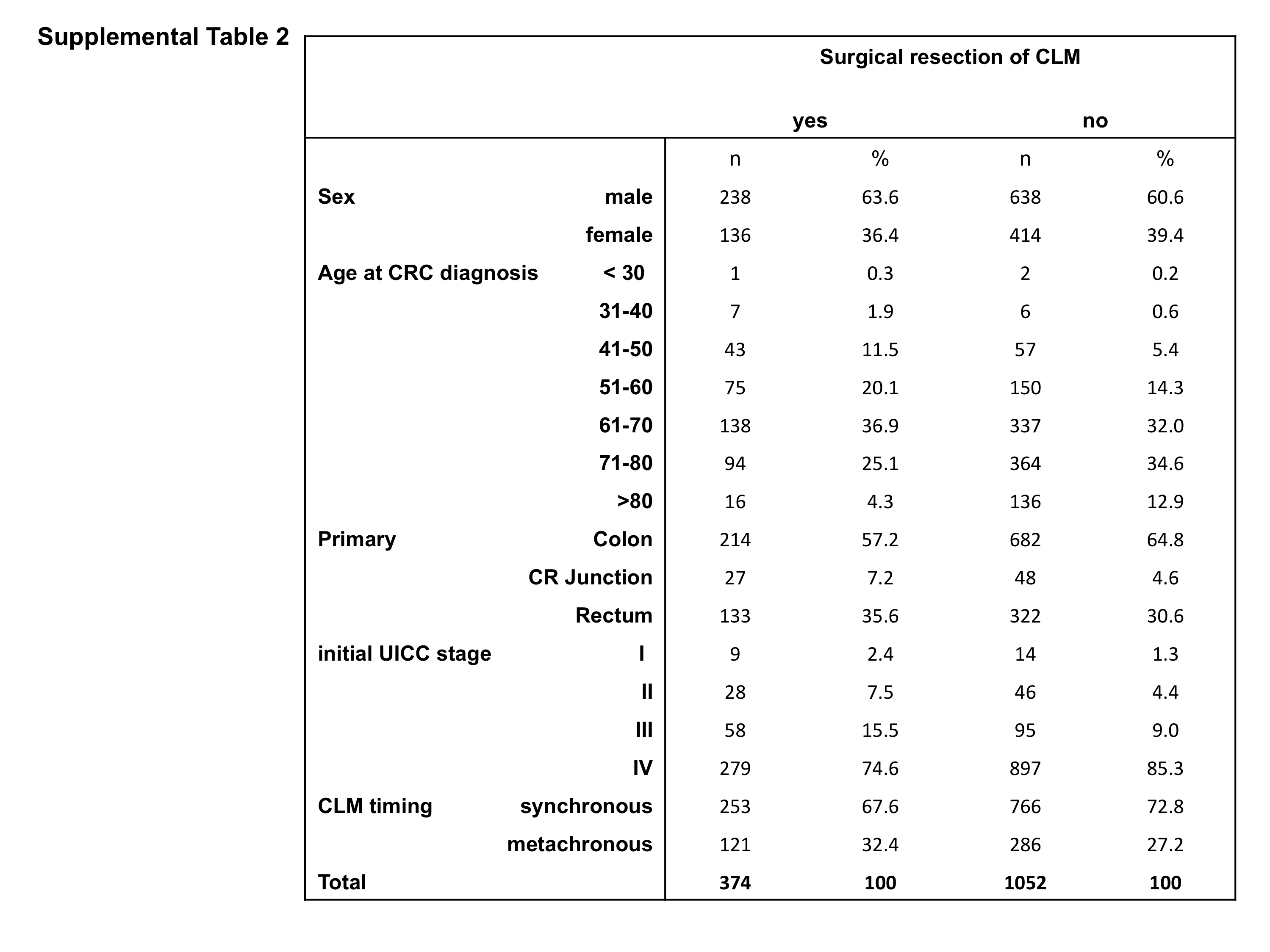

Supplement: Supplementary file 2 — Additional file 2: Table S2: Characteristics of 1426 colorectal liver metastasis (CLM) patients with or without curative liver resection. (TIFF 462 KB) [file 12885_2014_4981_MOESM2_ESM.tiff]

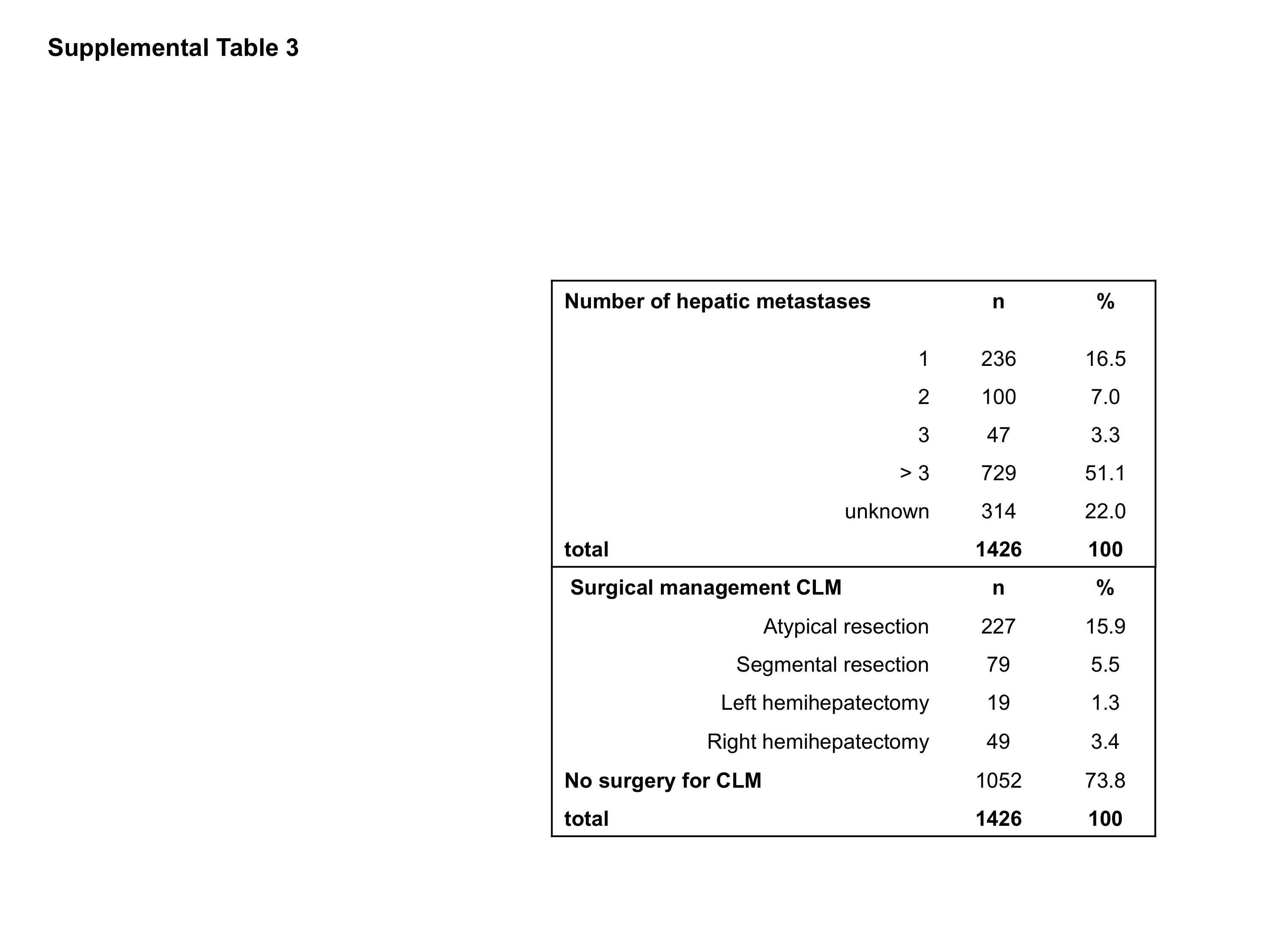

Supplement: Supplementary file 3 — Additional file 3: Table S3: Number of hepatic metastases and surgical management of 1426 patients with colorectal liver metastases. (TIFF 342 KB) [file 12885_2014_4981_MOESM3_ESM.tiff]

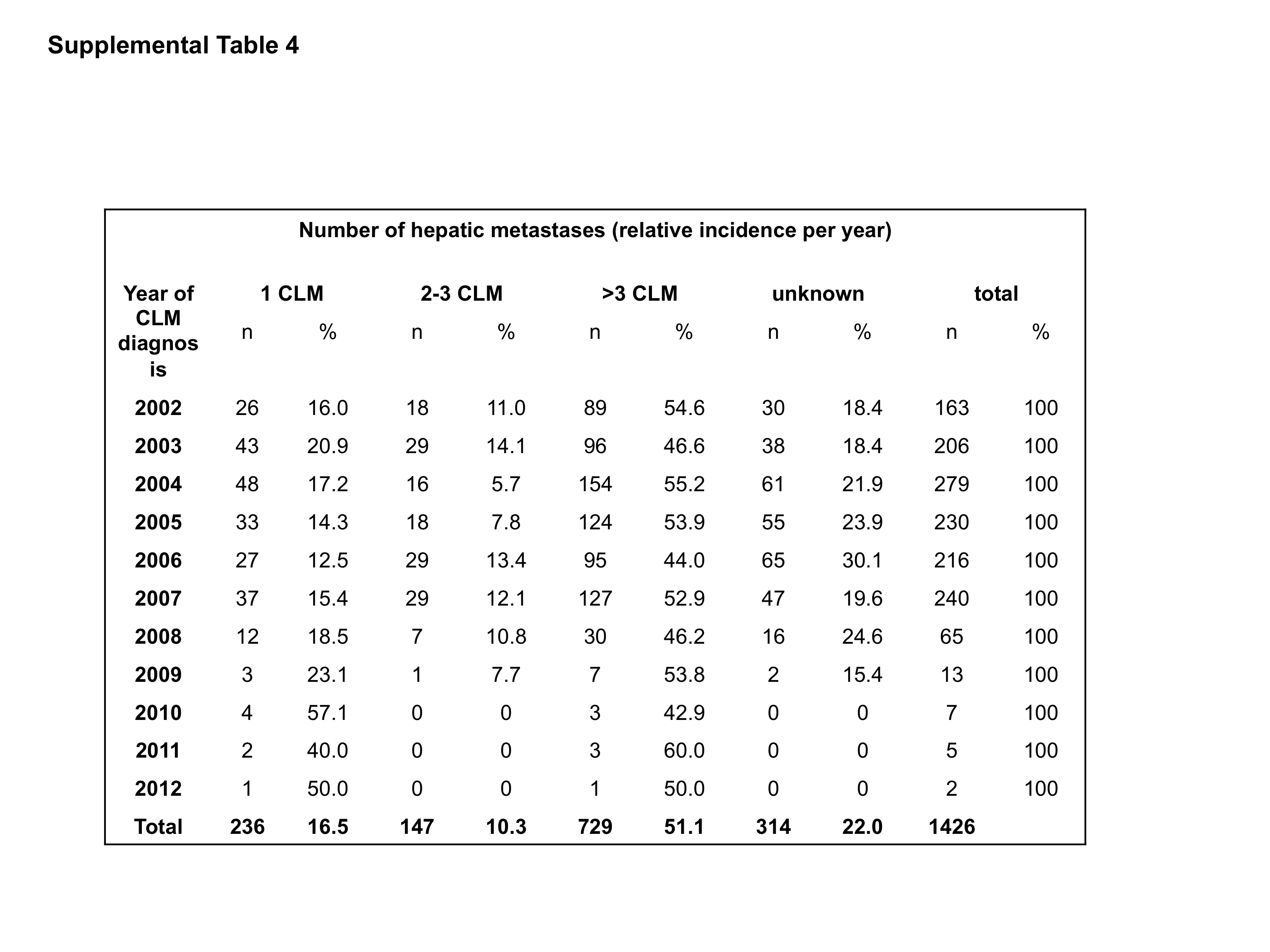

Supplement: Supplementary file 4 — Additional file 4: Table S4: Number of hepatic metastases diagnosed over time. (TIFF 463 KB) [file 12885_2014_4981_MOESM4_ESM.tiff]

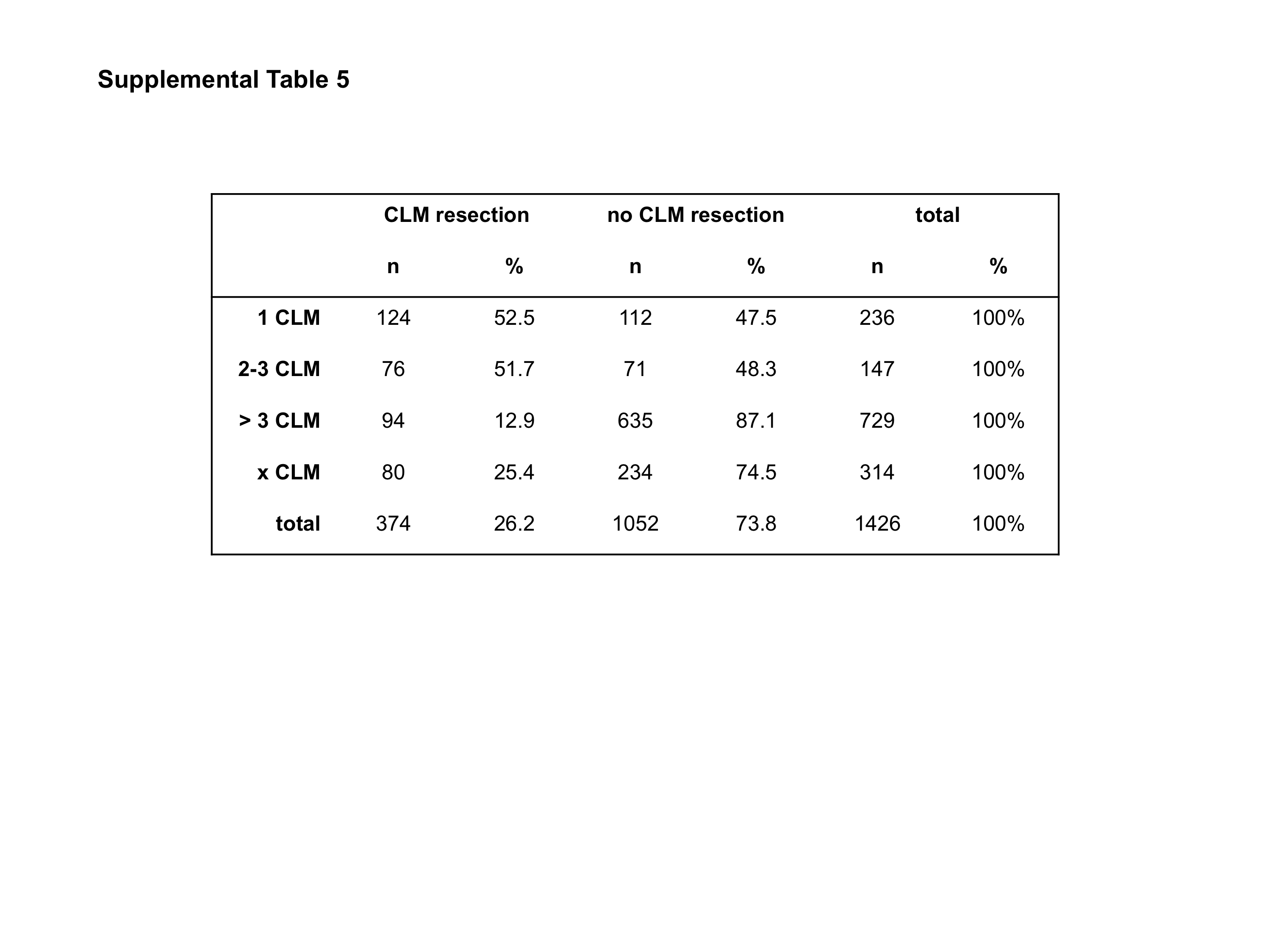

Supplement: Supplementary file 5 — Additional file 5: Table S5: Hepatic resection rates in relation to number of colorectal liver metastases. (TIFF 278 KB) [file 12885_2014_4981_MOESM5_ESM.tiff]

## Slide 1
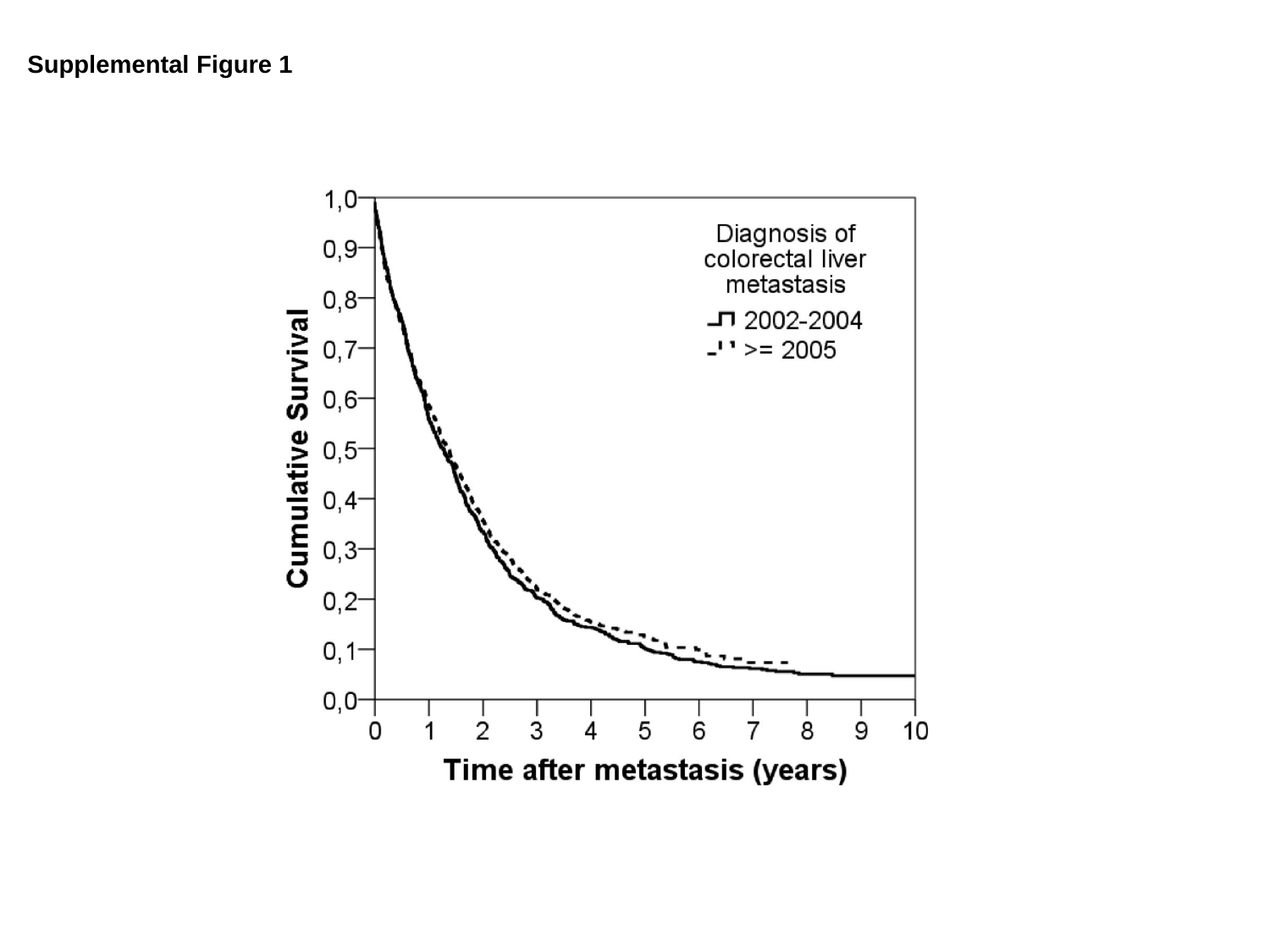

Supplemental Figure 1

Supplement: Supplementary file 6 — Additional file 6: Figure S1: Comparison of 10-year OS in patients diagnosed with colorectal liver metastases with and without liver metastases before and after 2005: A trend towards an improved survival in patients diagnosed 2005 and later was seen (not significant). (PPTX 264 KB) [file 12885_2014_4981_MOESM6_ESM.pptx]
